# Neuroprotective effects of a novel peptide from *Lignosus rhinocerotis* against 6‐hydroxydopamine‐induced apoptosis in PC12 cells by inhibiting NF‐κB activation

**DOI:** 10.1002/fsn3.3050

**Published:** 2022-09-12

**Authors:** Chuan Xiong, Yu Zhu, Qiang Luo, Chia Wei Phan, Yujie Huo, Ping Li, Qiang Li, Xin Jin, Wenli Huang

**Affiliations:** ^1^ Biotechnology and Nuclear Technology Research Institute Sichuan Academy of Agricultural Sciences Chengdu China; ^2^ The Second Affiliated Hospital Chongqing Medical University Chongqing China; ^3^ Mushroom Research Centre Universiti Malaya Kuala Lumpur Malaysia; ^4^ Department of Pharmaceutical Life Sciences Faculty of Pharmacy Universiti Malaya Kuala Lumpur Malaysia; ^5^ Yunnan Plateau Characteristic Agricultural Industry Research Institute Yunnan Agricultural University Kunming China; ^6^ College of Food and Biological Engineering Chengdu University Chengdu China

**Keywords:** *Lignosus rhinocerotis*, neuroprotective effect, oxidative stress, peptide

## Abstract

According to previous studies, oxidative stress is a leading cause of dopaminergic neuron death and may contribute to the pathogenesis of Parkinson's disease (PD). In the current study, we used chromatography of gel filtration to identify a novel peptide (*Lignosus rhinocerotis* peptide [LRP]) from the sclerotium of *Lignosus rhinocerotis* (Cooke) Ryvarden. Its neuroprotective effect was evaluated using an in vitro PD model constructed by 6‐hydroxydopamine (6‐OHDA)‐stimulated to apoptosis in PC12 cells. The molecular weight of LRP is determined as 1532 Da and the secondary structure is irregular. The simple amino acid sequence of LRP is Thr‐Leu‐Ala‐Pro‐Thr‐Phe‐Leu‐Ser‐Ser‐Leu‐Gly‐Pro‐Cys‐Leu‐Leu. Notably, LRP has the ability to significantly boost the viability of PC12 cells after exposure to 6‐OHDA, as well as enhance the cellular activity of antioxidative enzymes like superoxide dismutase (SOD), catalase (CAT), and glutathione peroxidase (GSH‐Px). LRP also lowers the level of malondialdehyde (MDA), decreases the activation performance of Caspase‐3, and reduces 6‐OHDA‐induced apoptosis via inhibition of nuclear factor‐kappa B (NF‐κB) activation. These data indicate that LRP may have the potential to act as a neuroprotective agent.

## Graphical abstract

In this study, a novel peptide (LRP) was extracted from the fruiting bodies of L. rhinocerotis using gel filtration chromatography. We determined the amino acid sequence of LRP, which was conducive to its artificial synthesis. Moreover, with NF‐κB as the target, the neuroprotective function of LRP was explored. LRP may have the potential to act as a neuroprotective agent.

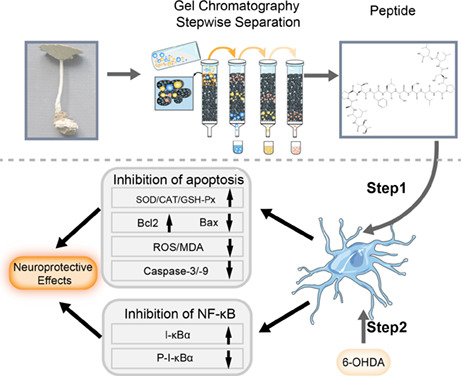

## 1 INTRODUCTION

Parkinson's disease (PD) affects 2%–3% of individuals over the age of 65, and it is regarded as the second most common neurodegenerative disease in humans (Darvas et al., 2014). Pathologically, PD is often characterized by a significant decrease of dopaminergic neurons in the substantia nigra, which leads to a reduction in dopamine biosynthesis in both the substantia nigra and striatum (Tysnes & Storstein, 2017). The mechanism of this neuronal degeneration is unclear, although many factors may contribute to this pathology, including genetic factors, environmental influences, aging, and oxidative stress (Burbulla et al., 2017). Among these, oxidative stress is considered an important cause of PD (Fahn & Cohen, 1992). Although normal physiological activities of the human brain require lots of oxygen, antioxidant enzymes are relatively scarce, making the brain particularly vulnerable to oxidative stress‐related damage (Lau et al., 2007). Indeed, prior research has demonstrated that the increase of mitochondrial oxidative stress in substantia nigra compacta dopaminergic neurons triggers dopamine‐dependent toxic cascade reactions that result in lysosomal dysfunction and α‐synuclein accumulation—the two main pathological features of PD (Kalia & Lang, 2015; Vandiver et al., 2013). Conventional dopamine replacement therapy (such as L‐3,4‐Dihydroxyphenylalanine [L‐DOPA]) is the most widely prescribed medicine for the treatment of PD symptoms, but has significant side effects (Kwon et al., 2010) including reduced motor function in the patients who have undergone long‐term L‐DOPA treatment (Maharaj et al., 2005). This limitation prompted us to identify new neuroprotective agents to prevent and treat PD. Since previous research suggests that oxidative stress contributes to the pathogenesis and progression of PD (Flower et al., 2005; Guo et al., 2007), we investigated whether treatment aimed at reducing oxidative stress may help slow the progression of PD.

Nuclear factor‐kappa B (NF‐κB) regulates a variety of physiological activities, including tumorigenesis (Rayet & Gélinas, 1999), and inflammation (Oseguera‐Toledo et al., 2011), immune response (Xu et al., 2016), and cell apoptosis (Habens et al., 2005). When a trigger (such as tumor necrosis factor α [TNF‐α] or inflammatory factors) does not exist, NF‐κB is sequestered in the cytoplasm by the inhibitory subunit I kappa B (I‐κB) protein (De Simone et al., 2015). After stimulation, I‐κB phosphorylation and degradation promotes nuclear membrane translocation. Indeed, NF‐κB enters the nucleus and activates regulatory activities (Baeuerle & Henkel, 1994). Furthermore, cellular oxidative stress activates NF‐κB (Panet et al., 2001). This activation initiates pro‐apoptotic gene expression that may be crucial to the onset and progression of neurodegenerative diseases (Hunot et al., 1997). Thus, NF‐κB could be a target for some substances (such as resveratrol, artemisinin; Seo et al., 2018) that have neuroprotective attributes.

Natural resource extraction of chemicals favorable to human nervous system health has made significant progress in recent decades (Martorell et al., 2016; Sato et al., 2022). Indeed, Salidroside, isolated from the root of *Rhodiola rosea*, protects apoptotic PC12 cells by inhibiting oxidative stress and inflammation (Zhou et al., 2019), and Neoechinulin A, an alkaloid from *Eurotium rubrum*, protects neuronal PC12 cells from peroxynitrite cytotoxicity (Kimoto et al., 2007). Furthermore, paeoniflorin (a compound found in *Paeonia lactiflora*) inhibits NF‐κB activation and prevents 6‐hydroxydopamine (6‐OHDA)‐induced cell death in PC12 cells (Cao et al., 2010). However, compared to plants, there are few studies on fungal neuroprotective agents. While some fungal polysaccharides have free radical scavenging activity that may play a neuroprotective effect (Giavasis, 2014), fungal polysaccharides have large molecular weights and complex structures, which make it difficult to penetrate the blood–brain barrier. Thus, their application as neuroprotective agents is limited. Nevertheless, ethanol or water extract of some fungi contains neuroprotective factors (Xiong et al., 2017). However, the components of the extracts are not clear, and the structure of functional substances needs to be identified.

The “Tiger Milk Fungus,” or *Lignosus rhinocerotis* (Cooke) Ryvarden, is a Polyporaceae mushroom that is frequently used in Malaysian traditional medicine to increase stamina, reduce cough, and help with asthma treatment (Sabaratnam et al., 2013). Recent scientific studies have confirmed the pharmacological benefits of *L. rhinocerotis* and revealed its antioxidant, antitumor, antiviral, and immunomodulatory activities (Nallathamby et al., 2018). Notably, the neuritogenic properties of *L. rhinocerotis* promote neurite outgrowth in PC12 cells: when applied to PC12 cells, the aqueous extract of *L. rhinocerotis* increased the population of neurite‐bearing cells from 9.8% to 23.6% (Eik et al., 2012). However, the components in the aqueous extract are complex, and it is difficult to determine which substance or substances may be responsible for the observed neuritogenic effects.

The PC12 cell line is derived from the adult rat adrenal medullary pheochromocytoma. The cell has a reversible neuronal phenotype response to nerve growth factor (NGF); it has been widely employed as a model to investigate dopamine biosynthesis and oxidative stress‐induced cytotoxicity (Rostamian Delavar et al., 2018). 6‐OHDA has also been widely used to create models of PD by reactive‐oxygen species (ROS)‐generated damage to dopaminergic neuronal cells (Deumens et al., 2002). Recently, a novel peptide (*L. rhinocerotis* peptide [LRP]) was extracted from the sclerotium of *L. rhinocerotis* and purified using reverse‐phase high‐performance liquid chromatography (RP‐HPLC) after being separated via molecular exclusion chromatography; its sequence was determined using liquid chromatography–tandem mass spectrometry (LC–MS–MS). The function of LRP for 6‐OHDA‐induced PC12 cell apoptosis and the potential pathway of LRP using NF‐κB as a key target was also investigated.

## 2 MATERIALS AND METHODS

### 2.1 Materials and chemicals

The sclerotial powder of *L. rhinocerotis* was contributed by Prof. Dr. Vikineswary Sabaratnam from the Mushroom Research Centre of Universiti Malaya. The reagents used to extract, separate, and purify peptides (including Brownlee analytical C18 column and Sephadex 30 Increase column) were purchased from PerkinElmer (Shelton, USA) and GE Healthcare (Uppsala, Sweden), respectively. The American Type Culture Collection (ATCC) provided the rat pheochromocytoma (PC12) cell line for use in this study (#CRL‐1721). Cell culture (RPMI [Roswell Park Memorial Institute] medium 1640, fetal bovine serum [FBS], horse serum [HS], and trypsin–EDTA [ethylenediaminetetraacetic acid]) was acquired from Gibco (Grand Island, NY, USA).

Nerve growth factor‐7S (NGF‐7S), 6‐OHDA, and 3‐[4, 5‐dimethylthiazol‐2‐yl]‐2, 5‐diphenyltetrazolium bromide (MTT) were acquired from Sigma (St. Louis, MO, USA). Primary antibodies (Caspase‐3, cleaved Caspase‐3, and glyceraldehyde‐3‐phosphate hydrogenase [GAPDH]) were obtained from Cell Signaling Technology (Danvers, MA, USA) for use in the Western blot analyses. Commercial kits were utilized to determine the activity of endogenous antioxidant enzymes (catalase [CAT], superoxide dismutase [SOD], and glutathione peroxidase [GSH‐Px]) and were obtained from Beyotime (Shanghai, China) along with Caspase (Caspases‐3 and ‐9). The DCFDA/H2DCFDA‐Cellular ROS Assay Kit was obtained from Abcam (Cambridge, UK), and antibodies for NF‐κB, I‐κBα, and phospho‐specific I‐κBα were obtained from Cell Signaling (Beverly, MA, USA). Other chemical reagents were used analytically.

### 2.2 Segregation and purification of peptides

The *L. rhinocerotis* sclerotial powder was collected by the passage of the material through a 200‐mesh screen. Next, 100 g of powder was soaked in 2 L of phosphate‐buffered saline (PBS, pH = 7.2) and stirred with a magnetic stirrer for 6 h to form a slurry. The supernatant was then recovered by centrifuging the slurry at 5000 g for 10 min to remove any remaining particles. To obtain the precipitate, ammonium sulfate was added to the supernatant after the reaction was completed. The concentration of ammonium sulfate in the supernatant increased from 40% to 80%, and all precipitates were collected. The precipitates were then dissolved in 100 ml of distilled water. The precipitate aqueous solution was dialyzed (Union Carbide Corporation, Houston, TX, USA) to separate the solids from the liquid with a cutoff molecular weight of 3500 Da. To extract the mushroom crude protein, we then condensed (Eyela, Tokyo, Japan) and lyophilized (Christ, Osterode, Germany) the dialysate. Then, the mushroom crude protein extract was introduced to a Sephadex 30 increasing column (2.6 cm × 30 cm), which was eluted with five bed volumes of distilled water (DW) at a flow rate of 0.8 ml/min. A ultraviolet (UV) detector measured the eluate, which was collected in 2.5 ml sample tubes at 215 nm. The most concentrated peak of the protein content was extracted, after which it was purified by dialysis and lyophilization to create the mushroom protein extract.

Reverse‐phase high‐performance liquid chromatographywas utilized to further purify the mushroom protein extract. First, 10 ml of distilled water (DW) was added to 100 mg of protein extract to yield a solution with a concentration of 10 mg/ml. Then, a PerkinElmer (Shelton, USA) N2600580 system with a C18 column set at 25°C for purification was used for RP‐HPLC analysis (injection volume of 20 μl; flow rate of 1 ml/min). The distilled water (A) and acetonitrile containing 1% trifluoroacetic acid (B) were set as mobile phase with a detection wavelength of 215 nm. The gradient elution was completed as follows: A: 0–8 min, 99%–97%; 8–12 min, 97%–96%; 12–16 min, 96%–80%; 16–20 min, 80%–99%. After tying up the radical rummaging task, the absorption peak was obtained, and the peptide extract was deemed appropriate for the current experiments because of its encouraging scavenging ability.

A Triple TOF 5600 LC/MS system was used to conduct the mass spectrometry analysis in this study (SCIEX, USA). Aspiration of the peptide sample was accomplished using a preprogrammed injector, which was subsequently combined with the C18 capture pillar (5 μm, 5 × 0.3 mm); the samples were then eluted and analyzed (75 μm × 150 mm, 3 μm fragment dimension, 100 Å pore expanse, Eksigent, CA, USA). The mobile stage included (A) distilled water trifluoroacetic acid solution (0.1%) and (B) acetonitrile trifluoroacetic acid solution (0.1%). The gradient elution was performed as follows: 0 min of 3% B, 0.1 min of 3%–7% B, 39.9 min of 7%–23% B, 3 min of 23%–50% B, 2 min of 50%–80% B, 5 min of 80% B, 0.1 min of 80%–5% B, and 5% B for 9.9 min. The flow rate of the liquid phase was synchronized at 300 nl/min. A single mass spectrometer full scan (with an *m*/*z* (mass‐to‐charge) range of 350–1500 and an ion buildup time of 250 ms) was included in each scan cycle during the information‐dependent acquisition (IDA) mass spectrometry portion of the study; this was followed by 40 MS/MS scans (with an *m*/*z* range of 100–1500 and an ion buildup time of 50 ms). MS/MS collecting parameters included a preceding ion warning greater than 120 cps (cycles per second) and a charge number between +2 and +5 for the duration of collection. ProteinPilot was used to obtain mass spectrum data (V4.5).

### 2.3 Morphology observation

To analyze the shape of the peptide, scanning electron microscopy (SEM) and atomic force microscopes (AFMs) were utilized. The peptide powder (2 mg) was secured to the SEM reinforcement table and sputter‐coated with gold using a vacuum‐based sputter coater (Q150R Plus; Quorum, UK). The peptide was then examined under a scanning electron microscope (Quanta FEG 250; Oregon, USA) to determine the quality. All samples were subjected to an accelerating voltage of 20.0 kV during the examination. Micrographs of the peptide were obtained at 20000× and 5000× magnification.

The peptide solution was diluted to a concentration of 1 mg/ml, then mixed with Milli‐Q water. Then, 10 μl of the diluted solution was placed on a freshly split mica platform and washed with Milli‐Q water to remove any remaining untarnished peptide residue. After air‐drying, the sample was scanned using an AFM straightaway (Hitachi SPM400, Japan).

### 2.4 Circular dichroism spectroscopy

To prepare the solution at our desired concentration of 0.25 mg/ml, we dissolved the peptide in water. The circular dichroism (CD) spectra measurement was performed on a Chirascan V100 (Applied Photophysics Limited, Surrey, UK) at 25°C using a 2 mm quartz cuvette (Qiu et al., 2009). The CD spectra were collected from 190 to 260 nm three times for the average at 1 nm intervals and 1 nm bandwidths.

### 2.5 Cell culture and treatment

PC12 cells were planted in the environment of RPMI medium 1640 supplemented with 5% FBS, 10% horse serum, 100 μg/ml streptomycin, and 100 U/ml penicillin in an incubator at 37°C with 5% carbon dioxide for 2 days. PC12 cells were then induced to differentiate with nerve growth factor beta (NGF‐β) at a final concentration of 50 ng/ml for 7 days. Afterward, cells were pretreated with LRP at different concentrations (10, 20, 40, 80, and 160 μM) for 6 h, followed by 6‐OHDA.

### 2.6 Cell viability assay

The MTT assay was used to evaluate the cytoprotective efficacy of LRP against cell damage produced by 6‐OHDA. Each well was filled with 10 μl MTT solution (5 mg/ml in PBS) and 90 μl FBS‐free media after collection of the supernatant. To deliquesce the formazan, 150 μl of dimethyl sulfoxide (DMSO) was added 4 h after MTT was applied. The rate of absorption of each well was then detected at 550 nm using a microplate reader (SpectraMax Plus 384; Molecular Devices, San Jose, CA, USA). Cell viability was relatively determined by the controls.

### 2.7 Reactive oxygen species measurement

A DCFDA/H2DCFDA‐Cellular ROS Assay Kit was used to measure cellular ROS. Briefly, cells were washed twice with prewarmed PBS in a 96‐well plate after drug treatment. Forty‐five minutes of exposure to DCFDA was used to stain the cells. After washing, absorbance was detected at 535 nm in a microplate reader (SpectraMax plus 384; Molecular Devices, San Jose, CA, USA). In the control group, the amount of ROS was normalized to the amount of generated fluorescence.

### 2.8 Determination of malondialdehyde (MDA) content and antioxidant enzymes' activity

*Lignosus rhinocerotis* peptide (of varying concentrations) was applied to PC12 cells for 6 h, after which the cells were treated with 6‐OHDA (100 μM) for 24 h. Following a PBS wash, cells were collected, exposed to 50 μl lysate, and centrifuged for 10 min at 12,000 g to obtain the supernatant for subsequent determination. MDA measurement was performed according to manufacturer's instructions using the 2‐thiobarbituric acid (TBA) technique (Beyotime, Shanghai, China). A commercial kit was then used to measure the endogenous antioxidant enzymes superoxide dismutase (SOD), catalase (CAT), and glutathione peroxidase (GSH‐Px) activity (Beyotime, Shanghai, China).

### 2.9 Apoptosis analysis

The apoptosis of PC12 cells was detected using flow cytometry. In brief, PC12 cells were treated with 6‐OHDA, digested with trypsin, and stained for 15 min with Annexin V/propidium iodide (PI). A FACSCalibur Flow Cytometer (BD, San Jose, CA, USA) was then used to analyze the cells at 488 nm wavelength and 530 nm of discharge wavelength after ~10,000 occurrences.

### 2.10 Real‐time polymerase chain reaction

Total RNA from cells was obtained using Trizol reagent (Sigma‐Aldrich, St. Louis, MO, USA). RT‐PCR amplifications were completed using a SYBR Premix Ex Taq according to the manufacturer's instructions. The RT‐PCR operating conditions were as follows: 94°C for 8 min, then 30 cycles at 94°C for 20 s, 56°C for 50 s, and 72°C for 90 s. Incubation time was set for 3 min at 72 degrees Celsius. The results are expressed as a ratio of optimum density to the amount of GAPDH present. Primers included: Bax (F: 5′‐CTGCAGAGGATGATTGCTGA‐3′; R: 5′‐GAGGAAGTCCAGTGTCCAGC‐3′); Bcl‐2 (F: 5′‐ATCTTCTCCTTCCAGCCTGA‐3′; R: 5′‐TGCAGCTGACTGGACATCTC‐3′); and GAPDH (F: 5′‐CACTCACGGCAAATTCAACGGCA‐3′; R: 5′‐GACTCCACGACATACTCAGCAC‐3′).

### 2.11 Measurement of Caspase activity

The activities of Caspase‐9 and Caspase‐3 were determined using commercial colorimetric assay kits following the manufacturers' instructions. After coculture with 6‐OHDA, PC12 cells were harvested and suspended for 5 min in lysis buffer on ice. For Caspase‐3, the supernatant was collected via centrifugation and mixed gently after the addition of acetyl‐Asp‐Glu‐Val‐Asp p‐nitroanilide (Ac‐DEVD‐pNA, 2 mM). For Caspase‐9, acetyl‐Leu‐Glu‐His‐Asp p‐nitroanilide (Ac‐LEHD‐pNA, 2 mM) was added. The mixture was incubated at 37°C for 60 min and the absorbance was read at a wavelength of 405 nm using a Microplate Reader (Molecular Devices, San Jose, CA, USA).

### 2.12 Western blotting

PC12 cells were pretreated with LRP (40 μM) for 6 h, followed by 6‐OHDA (100 μM) for varying durations. A Nuclear and Cytoplasmic Protein Extraction Kit (Beyotime, Shanghai, China) was used to fractionate the nuclear and cytoplasmic components of cells following the manufacturer's instructions. Electrophoresis with 10% sodium dodecyl sulfate (SDS) and polyacrylamide gel electrophoresis (PAGE), followed by electrotransfer onto a polyvinylidene difluoride (PVDF) membrane (Thermo Fisher Scientific Inc., Waltham, MA, USA), was used to divide the obtained protein (20 μg). Then, the membranes were blocked and probed with main antibodies at 4°C overnight, after which they were incubated for an additional 1 h at 37°C with horseradish peroxidase (HRP)‐conjugated secondary antibodies. Using a chemiluminescence tracking system (Bio‐Rad, Hercules, CA, USA), target protein bands were visualized and subsequently measured using densitometric analysis (Quantity One software, Bio‐Rad, Hercules, CA, USA).

### 2.13 Statistical analysis

All results are expressed as mean ± standard deviation (*SD*) of triplicate values. One‐way analysis of variance (ANOVA) was applied to determine significant differences between the groups using SPSS 19.0 (SPSS Inc., Chicago, IL, USA). Significance was assigned at *p* < .05 by Newman–Keuls tests (Duncan's Multiple Range Test [DMRT]).

## 3 RESULTS

### 3.1 Isolation and purification of LRP

**FIGURE 1:**
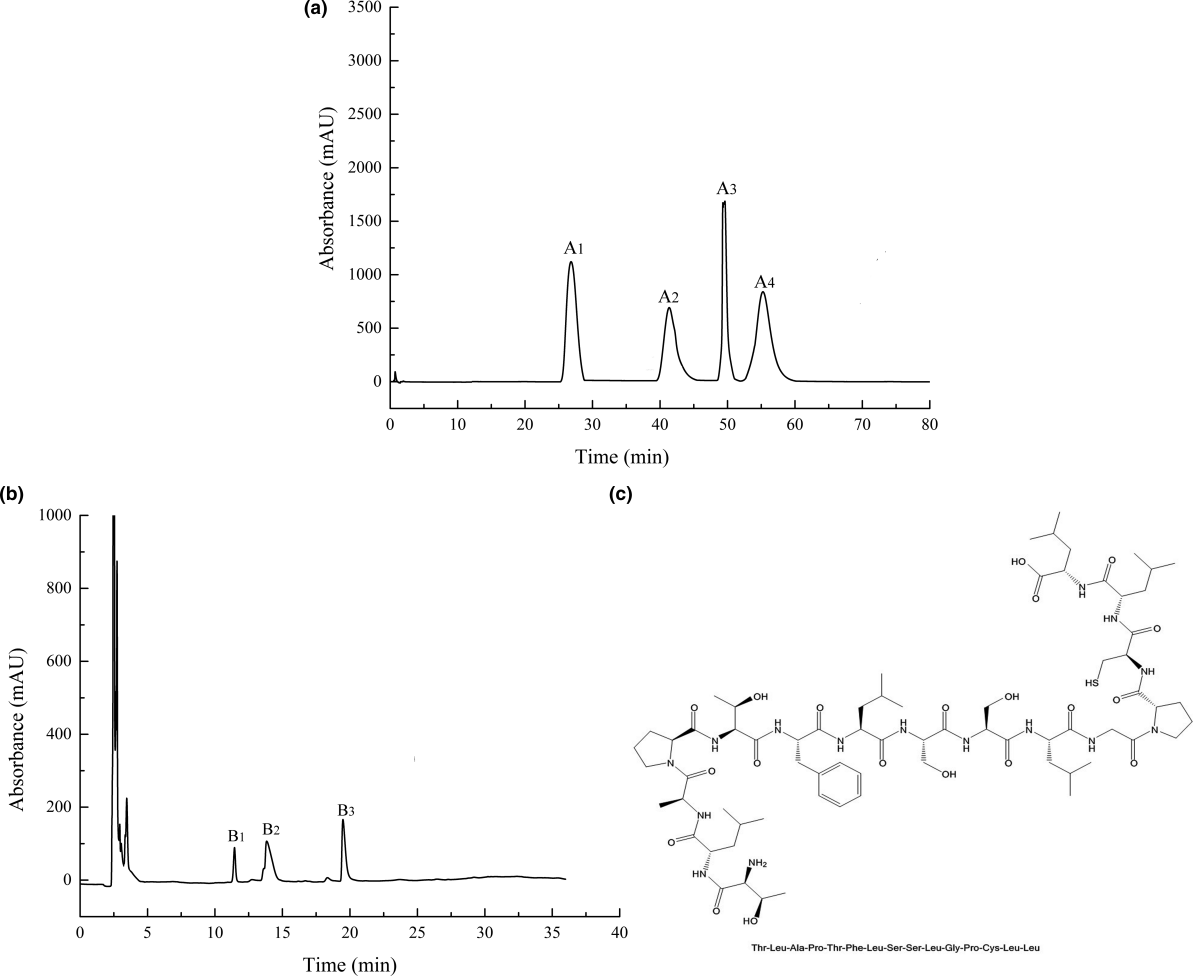
Isolation and purification of *Lignosus rhinocerotis* peptide (LRP). (a) Gel chromatography; (b) Reverse‐phase high‐performance liquid chromatography (RP‐HPLC); (c) Amino acid sequence of LRP. The crude protein extract of *L. rhinocerotis* was purified by using ammonium sulfate precipitation. Purification of the crude protein extract was accomplished through the use of Sephadex 30 chromatography, which yielded four characteristic absorption peaks (A_1‐4_). The A_4_ component was further separated using RP‐HPLC, resulting in the separation of three fine components (B_1‐3_). To evaluate the amino acid composition of LRP, the B_2_ component was subjected to a further round of analysis using liquid chromatography–tandem mass spectrometry (LC–MS–MS).

The protein extract was subjected to stepwise ammonium sulfate precipitation followed by centrifugation to remove impurities. After freeze‐drying, the protein extract (9.82 g) was obtained from 100 g of dry powder of *L. rhinocerotis*. The extract was separated and fractionated using a Sephadex 30 column (2.6 cm × 30 cm), and then washed with distilled water to obtain a crude protein extract (Figure 1a). After three exposures to bed column volume, four elution peaks (A_1‐4_) were obtained. There was a mass of 1.92 g for the A_1_ component, and 1.58, 0.98, and 2.34 g for the A_3‐4_ components, respectively. Based on the appearance time of the characteristic peak, A_4_ was selected as the protein extract. The protein extract was further separated by RP‐HPLC (Figure 1b), and three characteristic absorption peaks were identified (B_1‐3_). One of them, B_1_, weighed 0.45 g; the others, B_2‐3_, weighed 0.62 and 0.58 g, respectively. According to the HPLC analysis, the purity of B_1‐3_ was more than 95%, with B_1_ having a purity of 97.42%, B2 having a purity of 97.04%, and B3 having a purity of 95.48% (Detailed test methods can be found in the supporting information). The B_2_ component had the best free radical scavenging activity (data not shown) and was therefore selected for LC–MS–MS analysis and subsequent harvest of the *L. rhinocerotis* peptide (LRP). LRP had a relative molecular weight of 1532 Da and contained 15 amino acids. The amino acid sequence was Thr‐Leu‐Ala‐Pro‐Thr‐Phe‐Leu‐Ser‐Ser‐Leu‐Gly‐Pro‐Cys‐Leu‐Leu.

### 3.2 Morphology observation

**FIGURE 2:**
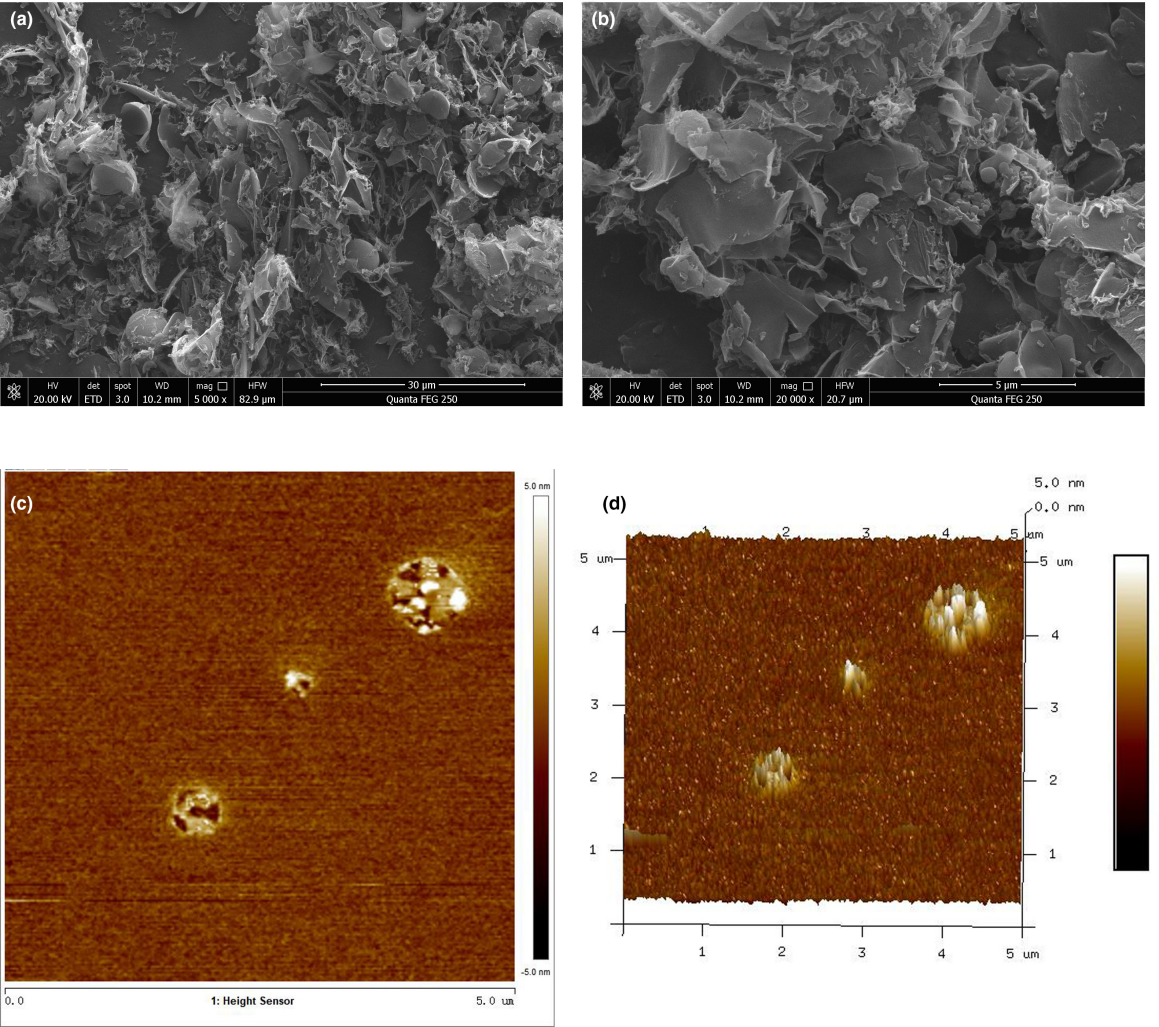
Morphology of *Lignosus rhinocerotis* peptide (LRP). (a and b) Scanning electron micrograph (SEM) images, 5000× and 20,000×, respectively; (c and d) Atomic force microscopy (AFM) image, c for the two‐dimensional (2D) image, and d for the three‐dimensional (3D) image.

*Lignosus rhinocerotis* peptide had an uneven flaky structure (Figure 2a), with curls appearing at the edges (Figure 2b), as demonstrated by scanning electron microscopy (SEM). In an aqueous solution (10 μM), LRP presents as a tight, granular structure. Notably, in the present study, more than 90% of the particles measured between 3 and 4 nm in height, with the tip of the particle pointing upward (Figure 2D). The average height of the particle tip was 3.51 nm.

### 3.3 Secondary structure of LRP

**FIGURE 3:**
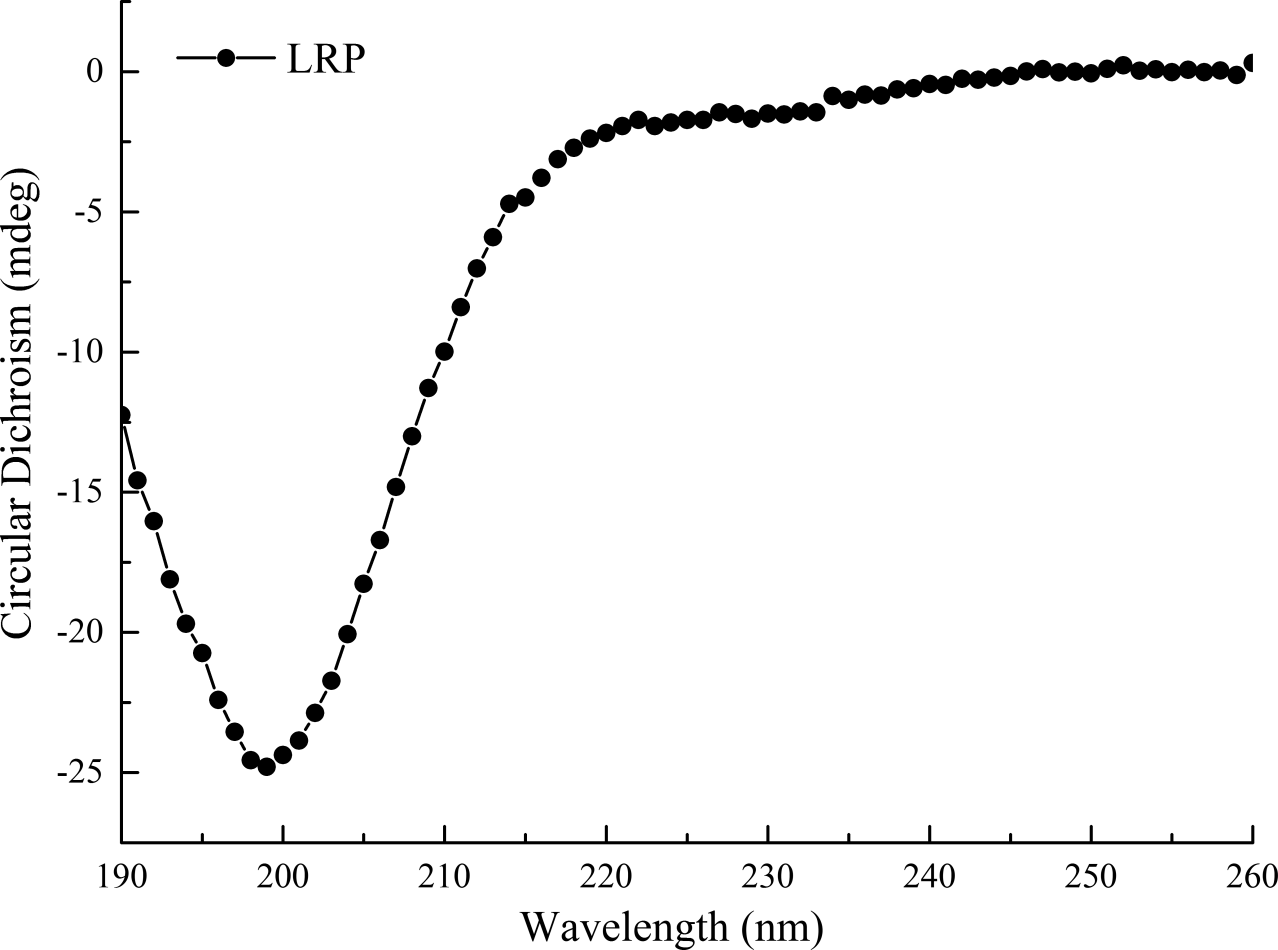
Circular dichroism (CD) spectra of *Lignosus rhinocerotis* peptide (LRP). In the vicinity of 198 and 210 nm, there were a strong negative band and a weak positive band, respectively, which indicates the presence of an irregular secondary structure for LRP.

The secondary structure of the LRP is shown in Figure 3. A strong negative band near 198 nm and a weak positive band near 220 nm were observed by far‐ultraviolet CD spectra; these bands are considered by some researchers to be characteristics of an irregular secondary structure (Liu et al., 2011). Further statistical analysis confirmed that 53.9% of LRP showed random coil and 34.6% showed β‐turn in aqueous solution.

### 3.4 Effect of LRP on viability of 6‐OHDA‐induced PC12 cells

**FIGURE 4:**
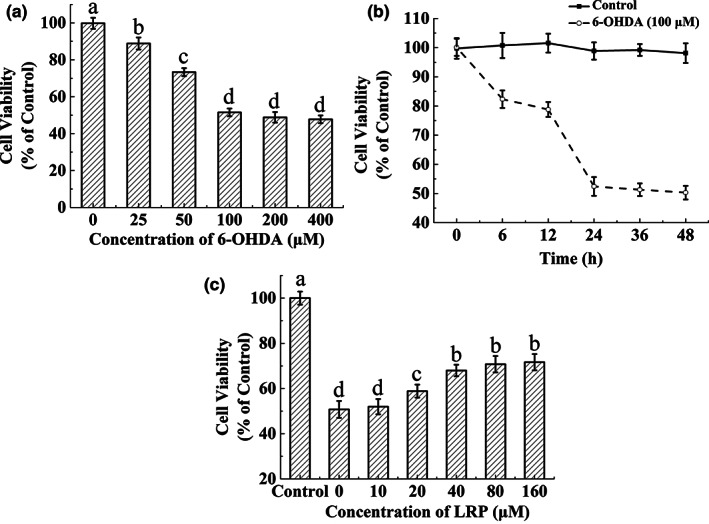
*Lignosus rhinocerotis* peptide (LRP) increased the cell viability of PC12 cells exposed to 6‐hydroxydopamine (6‐OHDA). (a) Cell viability determination was performed of PC12 cells cocultured with different concentrations of 6‐OHDA for 24 h. (b) Cell viability of PC12 cells cocultured with 6‐OHDA at a concentration of 100 μM for a different time. (c) In the presence or absence of 100 μM 6‐OHDA pretreatment for 24 h, the PC12 cells were treated with the specified dose of LRP to determine cell viability. In a and c, different lowercase letters represent significant differences at the 5% level. In panel B, *p < .05, **p < .01 compared with PC12 cells not exposed to 6‐OHDA.

*Lignosus rhinocerotis* peptide (0–160 μM) had no significant effect on PC12 cell viability (data not shown). However, 6‐OHDA had a significant inhibitory effect on the viability of PC12 cells in a concentration‐ and time‐dependent treatment manner (Figure 4a,b). After 24 h of treatment of 6‐OHDA (0–400 μM), the viability of PC12 cells decreased significantly (Figure 4a). Notably, when the concentration of 6‐OHDA reached 100 μM, the cell viability was only half that of the control group and did not decrease significantly with the continued increase in treatment concentration. Based on this result, the concentration of 6‐OHDA was set at 100 μM for each subsequent experiment. After 24 h of 100 μM 6‐ODHA and PC12 cell co‐incubation, vitality decreased (52.42 ± 2.97%). Continuation of incubation did not significantly alter the vitality. We then studied the effect of LRP on 6‐OHDA‐induced cytotoxicity (Figure 4c). MTT cell viability assays show that pretreatment with LRP (10–40 μM) significantly protects PC12 cells from 6‐OHDA‐induced damage in a dose‐independent manner. However, when the concentration exceeded 40 μM, there was no significant increase in cell protection (*p* > .05). Hence, 40 μM LRP was used in subsequent experiments.

### 3.5 LRP attenuated cellular oxidative stress damage in 6‐OHDA‐exposed PC12 cells

**FIGURE 5:**
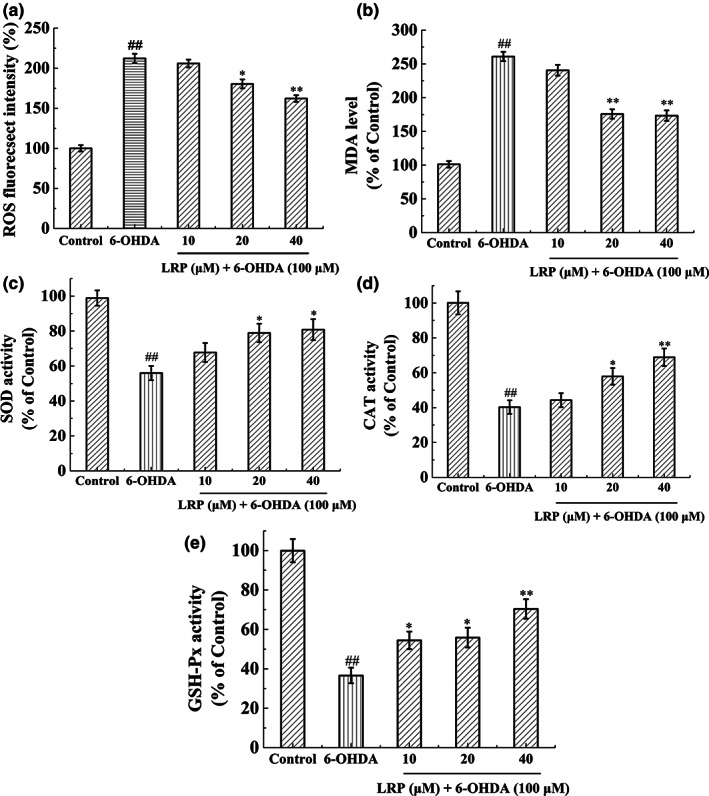
The effect of *Lignosus rhinocerotis* peptide (LRP) on 6‐hydroxydopamine (6‐OHDA)‐induced oxidative stress and endogenous antioxidant enzymes was investigated in PC12 cells. (a). The oxidant‐sensitive fluorescent probe 2,7‐dichlorodihydrofluorescein diacetate (DCFH‐DA) was used to monitor the generation of reactive oxygen species (ROS). (b) The effect of LRP on lipid peroxidation. (c–e) The effects of LRP on the activities of superoxide dismutase (SOD), catalase (CAT), and glutathione peroxidase (GSH‐Px), respectively. The percentage was used to express the change compared with the control group and the bar represents the mean ± *SD* (*n* = 3), ^##^ *p* < .01 compared with the control group. *p < .05, **p < .01 compared with the 6‐OHDA group.

The relative content of ROS allowed us to predict the strength of oxidative pressure. ROS in the PC12 cells that were exposed to 6‐OHDA had a marked increase (*p* < .01), compared to the control group (Figure 5a). However, pretreatment with different concentrations (20 and 40 μM) of LRP significantly reduced both the production of ROS and cellular malondialdehyde (MDA) content in PC12 cells (Figure 5a,b). Notably, ROS induces lipid peroxidation (which ultimately produces MDA), and the MDA content of 6‐OHDA‐treated PC12 cells was significantly higher (260.92 ± 12.6%) than the control group (98.89 ± 2.24%, *p* < .01). However, the content of MDA dropped by ~30% to 175.39 ± 6.92% and 173.33 ± 7.89% after pretreatment with 20 or 40 μM LRP, respectively. The activity of endogenous antioxidant enzymes, which include superoxide dismutase (SOD), catalase (CAT), and glutathione peroxidase (GSH‐Px), was also significantly reduced in 6‐OHDA‐treated cells (55.9 ± 4.11%, 40.31 ± 3.86%, and 36.62 ± 3.96% of the control group, respectively). Hence, LRP dose‐dependently increased 6‐OHDA‐induced downregulation of SOD, CAT, and GSH‐Px.

### 3.6 Effects of LRP and 6‐OHDA on apoptosis of PC12 cells

**FIGURE 6:**
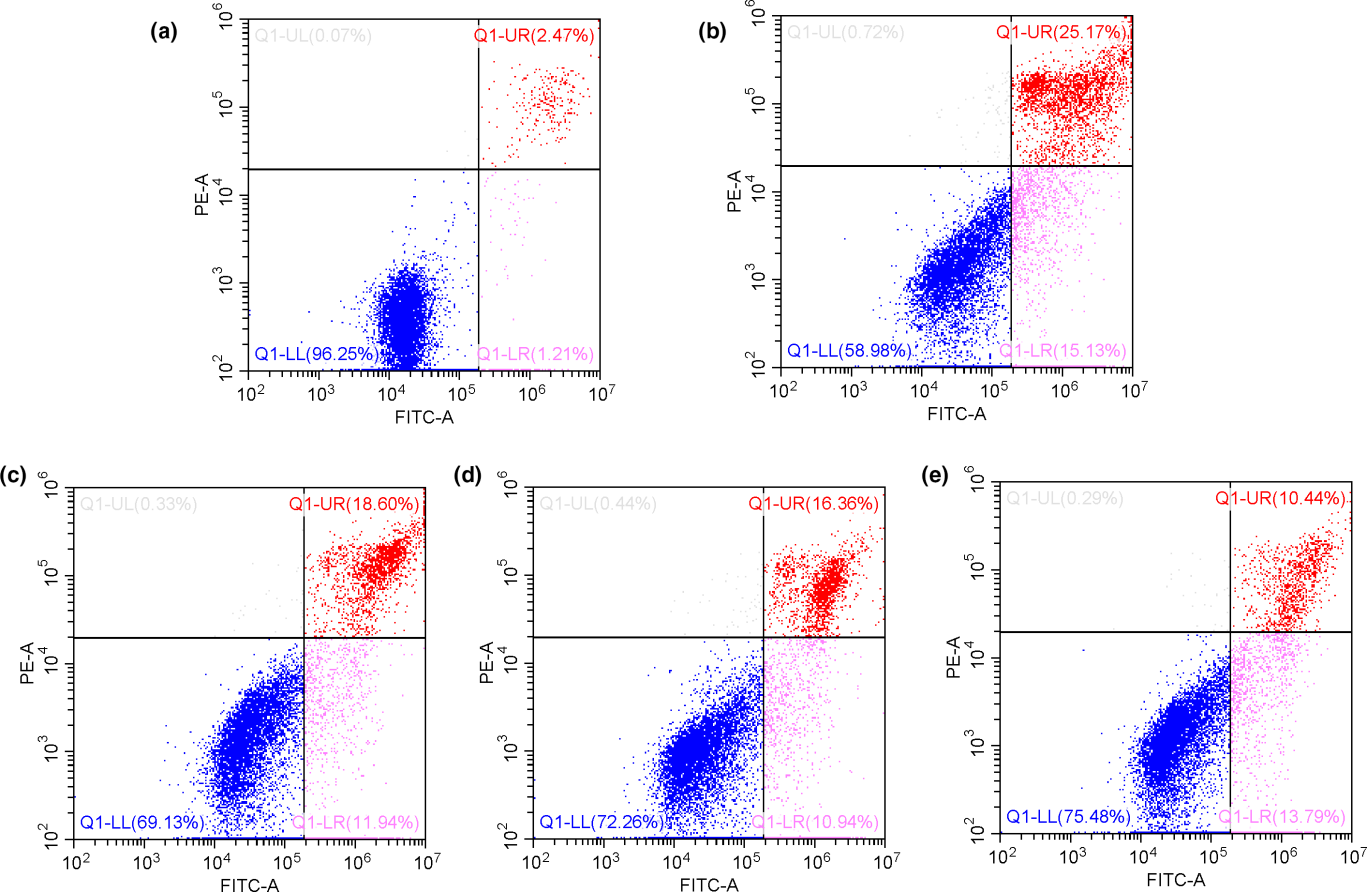
6‐Hydroxydopamine (6‐OHDA)‐induced PC12 cell apoptosis was assessed by flow cytometry using Annexin V/propidium iodide (PI) double staining. (a) Control (untreated) cells; (b) Cells cultured with 6‐OHDA (100 μM); (c–e) Cells treated with 10, 20, and 40 μM of *Lignosus rhinocerotis* peptide (LRP) before they were exposed to 100 M 6‐OHDA for 24 h, respectively.

After 6‐OHDA treatment, the ROS content in PC12 cells increased significantly (Figure 5a), and the resulting oxidative stress induced cell apoptosis (Figure 6a). However, the apoptotic rate of cells that were incubated with LRP before 6‐OHDA treatment was significantly reduced (Figure 6b–d).

**FIGURE 7:**
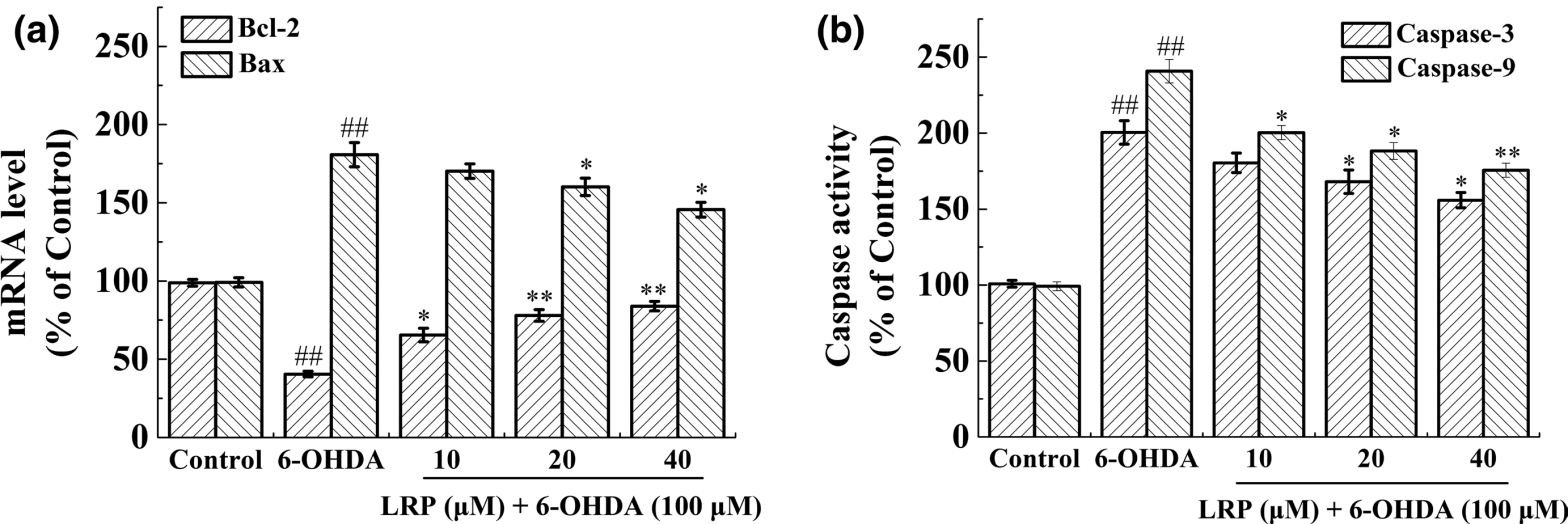
Effect of *Lignosus rhinocerotis* peptide (LRP) on apoptosis induced by 6‐hydroxyamine (6‐OHDA) in PC12 cells. (a) Detection of messenger RNA (mRNA) levels of Bax and Bcl‐2 was done by real‐time polymerase chain reaction (RT‐PCR). (b) The effect of LRP on the activities of Caspase‐3 and Caspase‐9 in PC12 cells that had been exposed to 6‐OHDA. Various doses of LRP were used to pretreat cells for 6 h before they were exposed to 100 M 6‐OHDA for 24 h. The bar represents the mean ± *SD* (*n* = 3) and ^##^ *p* < .01 compared with the control group, *p < .05, **p < .01 compared with the 6‐OHDA group.

Furthermore, 6‐OHDA treatment altered the ratio of B‐cell lymphoma 2:Bcl‐2 associated protein (Bcl‐2:Bax) in the cell, which may act as a molecular switch that dictates whether apoptosis is initiated. Indeed, Bcl‐2 mRNA (messenger RNA) levels were significantly reduced to 40.48 ± 1.74% in PC12 cells after 6‐OHDA stimulation. In contrast, Bax mRNA levels increased to 180.82 ± 7.72% (Figure 7a); the resulting ratio of Bcl‐2:Bax was 0.22. Pretreatment with LRP (40 μM) further altered the ratio of Bcl‐2:Bax to 0.58, as Bcl‐2 expression levels increased, while Bax expression levels decreased. Furthermore, both Caspase‐3 and Caspase‐9 levels significantly increased after 6‐OHDA treatment (Figure 7b). However, adding 20 and 40 μM LRP before 6‐OHDA treatment decreased Caspase‐3 activity to 168.01 ± 7.69% and 155.86 ± 5.02%, respectively. Moreover, 10 and 40 μM LRP impressively regulated the vitality of Caspase‐9, and activity was significantly different following 6‐OHDA treatment (*p* < .05, *p* < .01, respectively).

### 3.7 Nuclear NF‐κB in 6‐OHDA‐treated cells

**FIGURE 8:**
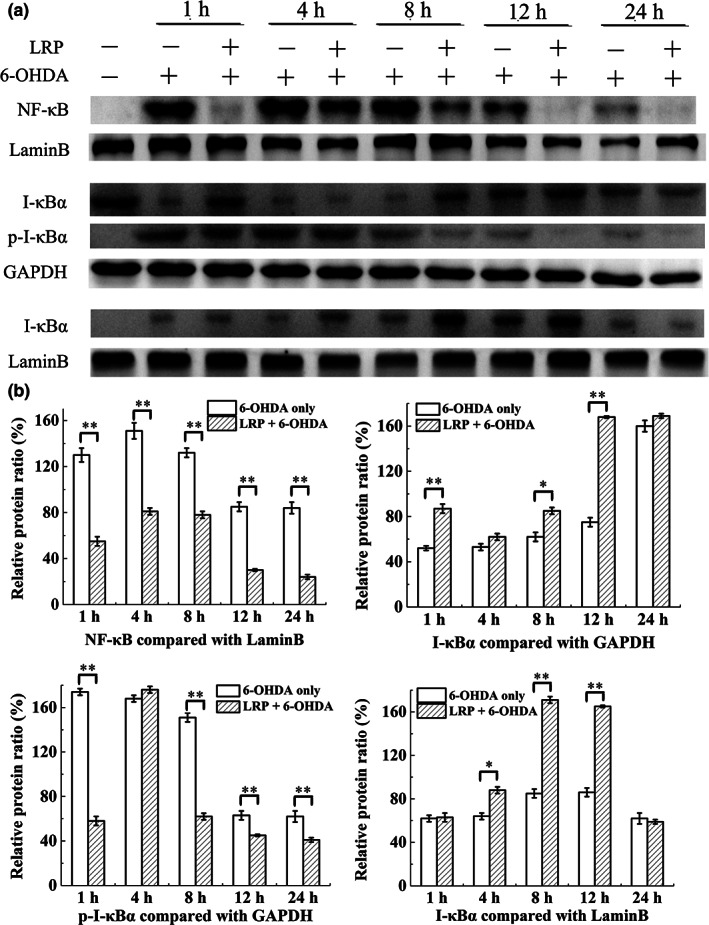
The mechanism of neuroprotective effects of *Lignosus rhinocerotis* peptide (LRP) on 6‐hydroxydopamine (6‐OHDA)‐induced PC12 cells. (a) The expression of nuclear factor‐kappa B (NF‐κB), I‐κBα (nuclear factor of kappa light polypeptide gene enhancer in B‐cells inhibitor, alpha), and phosphorylated I‐κBα (p‐I‐κBα) protein was identified via Western blotting. (b) The intensities of relative protein levels were quantified from (a). The ratio represents the expression level as compared to the respective internal control protein (%). PC12 cells were pretreated with LRP with a concentration of 40 μM for 6 h, following with 6‐OHDA at 100 μM for different durations. The protein expression of NF‐κB and I‐κBα in the nucleus, as well as I‐κBα and p‐I‐κBα in cytoplasm was semiquantitatively analyzed by Western blotting. *p < .05, **p < .01 compared with the 6‐OHDA group.

Since ROS overproduction was induced by 6‐OHDA exposure, the content of NF‐κB was significantly increased—a change that occurred within 1 h after 6‐OHDA treatment. Furthermore, the amount of NF‐κB remained high for 12 h, although LRP pretreatment slowed NF‐κB activation and reduced overall content. Interestingly, 6‐OHDA treatment rapidly phosphorylated I‐κBα in the cytoplasm of PC12 cells and kept p‐I‐κBα levels heightened for ~8 h. Changes in the content of the p‐I‐κBα promoted the dissociation of I‐κBα, which in turn promoted the activation of NF‐κB. However, LRP treatment slowed the phosphorylation of I‐κBα, resulting in lower levels of cytoplasmic p‐I‐κBα. In the nuclear protein of normal PC12 cells, the content of I‐κBα was low, although it increased significantly beginning 4 h after LRP treatment and continuing until 12 h after treatment (Figure 8).

## 4 DISCUSSION

Mushrooms are high in protein and provide numerous medicinal benefits, making them a good source of natural active peptides for research and development. A polypeptide derived from *Pleurotus eryngii* mycelium (PEMP) has anticancer, antioxidant, and immunostimulatory activities (Sun et al., 2017). Cordymin, a peptide derived from the medicinal fungus, *Cordyceps sinensis*, exhibits neuroprotective effects on the rat ischemic brain, inhibits inflammation, and increases antioxidant activity related to pathological changes (Wang et al., 2012). Because of their simple structure, reduced immunogenicity, and ease of artificial synthesis, mushroom‐based peptides are a significant component of contemporary medication development. Ion‐exchange chromatography and gel filtration chromatography are commonly used to separate physiologically active peptides from protein hydrolysates. Ion‐exchange chromatography distinguishes substances based on the charge of ions and polar molecules, whereas gel filtration chromatography distinguishes substances based on the sizes of individual molecules. However, no standard method to extract peptides from mushrooms currently exists. Indeed, peptides extracted from mushrooms are typically separated using different techniques, which makes it challenging to promote and use mushroom peptides in a consistent way.

In contrast to the mushroom polysaccharide (which can be extracted using a standard approach), the extraction of mushroom peptides requires further investigation. Polysaccharides are one of the most representative fungal active substances from *L. rhinocerotis*, which have been proven by many researchers to have antioxidant, antitumor, anti‐inflammatory, and immune‐enhancing activities (Keong et al., 2016; Lee et al., 2014; Seow et al., 2017). However, in this study, we have isolated a novel peptide, LRP, and it was shown to contain no polysaccharides as determined by the phenol–sulfuric acid method. Hence, we confirmed that the peptide contributed to the anti‐apoptotic effect in PC12 cells in this study.

The degeneration and cell death of dopaminergic neurons in the substantia nigra and striatum lead to dopamine deficiency, collectively manifested PD. Thus, dopamine replacement therapy (such as L‐3,4‐Dihydroxyphenylalanine, L‐DOPA) is currently one of the best treatment methods for idiopathic PD and effectively masks or reduces disease symptoms. However, long‐term L‐DOPA treatment may be associated with adverse motor effects, particularly after the initial response phase. In addition, some researchers have detected 6‐OHDA in the urine of PD patients who have been taking L‐DOPA for extended periods (Kwon et al., 2010). Here, we found that the presence of 6‐OHDA induced cells to produce ROS and cause oxidative damage, although long‐term L‐DOPA treatment produces more 6‐OHDA, which may negatively regulate PD treatment. Therefore, functional ingredients from natural products may aid in treating PD by removing active oxygen and relieving oxidative stress. According to this, peptides from Malaysian traditional medicinal fungi (*L. rhinocerotis*) were identified and purified for the preventive and adjuvant therapy of Parkinson's disease.

Paradoxically, eukaryotic aerobic creatures cannot survive without oxygen, but oxygen is fundamentally harmful to their survival (Davies, 1995). At the same time, the oxidation reaction provides energy, and superoxide anion radicals and hydroxyl radicals generated during the reaction cause oxidative damage. Endogenous antioxidant enzymes (among others) balance the production and consumption of ROS (Xiong et al., 2016). However, in elderly individuals, increased ROS production outpaces the antioxidative system's capacity to safeguard. Endogenous antioxidant enzymes (including SOD, CAT, and GSH‐Px) act as the body's natural resistance to the oxidative stress defense system (Liu et al., 2020). Following 6‐OHDA treatment, antioxidant enzyme activity in the cell significantly decreases. Hence, the antioxidant enzyme system in the cell exceeded the expected load. However, 6 h of LRP pretreatment significantly improved the endogenous antioxidant enzyme activity of PC12 cells, and the level of SOD reflected the load of the antioxidant enzyme system in the cell and indirectly indicates the level of free radicals in the cell. This result may indicate that pretreatment with LRP reduces the production of anionic superoxide. Furthermore, LRP pretreatment affected the metabolism of GSH‐Px (a critical component of cellular antioxidant defense and an electron donor for ROS) and reduced cell death induced by 6‐OHDA.

Reactive‐oxygen species easily damages biological molecules and ultimately causes cell death when the endogenous antioxidant enzyme system is unable to appropriately eliminate the ROS (Yoon et al., 2018). Bcl‐2 and Bax are the most representative apoptosis‐inhibiting genes and apoptosis‐promoting genes in the Bcl‐2 protein family. Notably, the Bax protein (produced by the Bax gene) forms a heterodimer with the Bcl‐2 protein and inhibits Bcl‐2. Researchers have reported that apoptosis is influenced by the ratio of Bax to Bcl‐2 proteins, and the degree of the inhibitory effect on apoptosis is a significant element in determining the strength of the effect itself (Zhang et al., 2019). Mechanistically, Bax induces apoptosis by entering mitochondria under the action of tBid (truncated Bid [BH3 interacting‐domain death agonist]), and increasing the permeability of the mitochondrial membrane prior to releasing cytochrome C. The release of cytochrome C is a critical step in the endogenous pathway of apoptosis, which occurs by targeting and activating Caspase‐9 (further leading to the activation of effector Caspases such as Caspase‐3; Shen et al., 2016). To evaluate the protective effect of LRP on 6‐OHDA‐induced apoptosis, we measured the expression levels of Bcl‐2 and Bax and the subsequent activity of Caspases‐3 and ‐9. Surprisingly, pretreatment of 6‐OHDA with LRP considerably enhanced Bcl‐2 levels and decreased Bax levels. Furthermore, Caspase‐3 and Caspase‐9 activities were both suppressed, suggesting that LRP may have an anti‐apoptotic effect by altering the proportion of Bcl‐2:Bax and Caspase‐3/‐9.

Continuous oxidative stress may cause NF‐κB to become active, and some researchers believe that the activation of NF‐κB is involved in the dopamine‐induced apoptosis of PC12 cells, which contributes to substantia nigra neurodegeneration in patients with PD (Sun et al., 2020). Here, we found that 6‐OHDA treatment quickly activated NF‐κB in PC12 cells. Specifically, NF‐κB in the cytoplasm significantly decreased, while NF‐κB in the nucleus increased rapidly in a short time. We also verified the mechanism of 6‐OHDA in activating NF‐κB through the classic activation pathway. In most cells, the NF‐κB/Rel transcription complex exists in the cytoplasm in an unactivated form bound to the inhibitor I‐κB. When the cell is stimulated (i.e., by ROS, as was the case in the current study), a series of phosphorylation and ubiquitination reactions lead to the degradation of I‐κB, and the NF‐κB transcription factor enters the nucleus (Zhang et al., 2020). In PC12 cells, 6‐OHDA treatment produces ROS, which further promotes the phosphorylation of I‐κB. After I‐κB dissociates, NF‐κB dimers enter the nucleus and bind to specific DNA sequences to regulate the expression of specific proteins that may affect cell apoptosis. Interestingly, we also observed that I‐κB phosphorylated and degraded in the cytoplasm rapidly re‐synthesized and entered the nucleus. We further confirmed that I‐κB binds to NF‐κB to form a complex of NF‐κB‐I‐κB, which causes NF‐κB to dissociate from its binding DNA κB site and retranslocate to the cytoplasm, completing the cycle of activation and inactivation of NF‐κB. In summary, pretreatment of LRP effectively inhibits phosphorylation of I‐κB, slows NF‐κB nuclear membrane translocation, and inhibits NF‐κB activation.

## 5 CONCLUSION

*Lignosus rhinocerotis* peptide has a strong inhibitory effect on 6‐OHDA‐induced apoptosis in PC12 cells. Indeed, LRP partially restores endogenous antioxidant enzyme activities, including SOD, CAT, and GSH‐Pxa. LRP also acts as an endogenous antioxidant enhancer by decreasing ROS levels and MDA generation. Besides, it also adjusts the ratio of Bax:Bcl‐2, inhibits the expression of Caspases‐9 and ‐3, and blocks mitochondrial pathway apoptosis. Moreover, LRP inhibits the activation of the NF‐κB pathway and attenuates oxidative stress injury induced by 6‐OHDA in PC12 cells. These findings show LRP's promise and lay the groundwork for more research into how it can be utilized to treat neurologic illnesses.

## CONFLICT OF INTEREST

The authors declare no conflict of interest.

## Supporting information


Appendix S1

## Data Availability

The original image of the target protein was uploaded as supporting information and all data can be obtained by contacting Dr. Pu Zhigang, the Head of Biotechnology and Nuclear Technology Research Institute (http://www.chinawestagr.com/swjshjsyjs/).
