# Supplementary material for: Neuroprotective effects of a novel peptide from Lignosus rhinocerotis against 6‐hydroxydopamine‐induced apoptosis in PC12 cells by inhibiting NF‐κB activation
Source: Food Sci Nutr. 2022 Sep 12;11(5):2152–65. doi: 10.1002/fsn3.3050 (PMC10171544; doi:10.1002/fsn3.3050)

S1, Component B purity measurement

The phase high performance liquid chromatography (HPLC) was used to determine the purity of B component. First, 1 mL of distilled water was added to 1 mg B_1_ to yield a solution with a concentration of 1 mg/mL. The injection volume was 14 μL and flow rate was 1 mL/min. The distilled water containing 0.1% trifluoroacetic acid (A) and acetonitrile containing 0.1% trifluoroacetic acid (B) were set as mobile phase included with a detection wavelength of 220 nm. Then a SHIMADZU (Kyoto, JPN) LC2020 system with a Inertsil ODS-SP(4.6*250 mm*5 μm) was used for analysis. The gradient elution was completed as follows: A: 0–20 min, 75%–35%; 20–30 min, 35%–0%; 30–38 min, 0%–0%; 38–40 min, 0%–75%. By comparing the peak area of the target peak, we determined that the purity of B_1_ component was 97.42%. The purity of B_2_ and B_3_ components was determined by the same method. The results showed that the purity of B_2_ component was 97.04%, and that of B_3_ component was 95.48%.


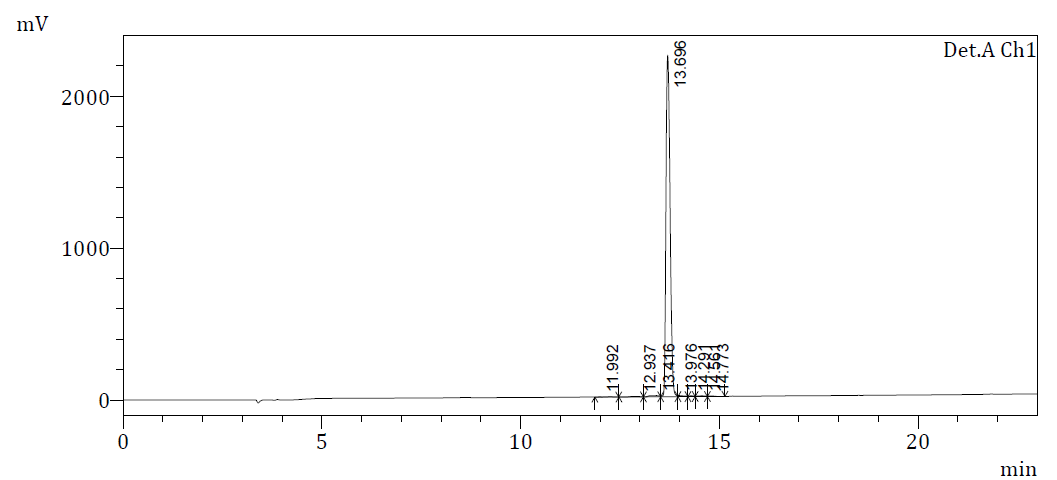


Fig. 1 HPLC chromatogram of B_1_ component

Table. 1 HPLC target peak information for B_1_ component

| Peak# | Ret. Time | Area | Height | Area % | Height % |
| --- | --- | --- | --- | --- | --- |
| 1 | 11.992 | 33279 | 2032 | 0.200 | 0.089 |
| 2 | 12.937 | 43304 | 2895 | 0.261 | 0.127 |
| 3 | 13.416 | 130695 | 7552 | 0.787 | 0.331 |
| 4 | 13.696 | 16169247 | 2248860 | 97.417 | 98.598 |
| 5 | 13.976 | 77132 | 6833 | 0.465 | 0.300 |
| 6 | 14.291 | 44140 | 4852 | 0.266 | 0.213 |
| 7 | 14.561 | 60750 | 4579 | 0.366 | 0.201 |
| 8 | 14.773 | 39388 | 3225 | 0.237 | 0.141 |
| Total |  | 16597933 | 2280827 | 100.00 | 100.00 |


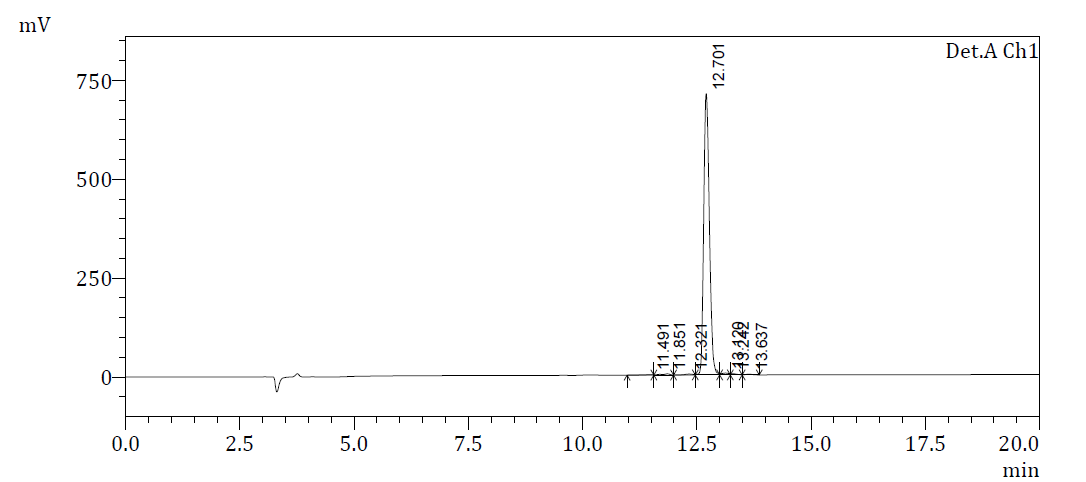


Fig. 2 HPLC chromatogram of B_2_ component

Table. 2 HPLC target peak information for B_2_ component

| Peak# | Ret. Time | Area | Height | Area % | Height % |
| --- | --- | --- | --- | --- | --- |
| 1 | 11.491 | 16197 | 990 | 0.256 | 0.136 |
| 2 | 11.851 | 37277 | 2854 | 0.588 | 0.393 |
| 3 | 12.321 | 30967 | 2397 | 0.489 | 0.330 |
| 4 | 12.701 | 6149840 | 711247 | 97.037 | 97.854 |
| 5 | 13.210 | 53653 | 4713 | 0.847 | 0.648 |
| 6 | 13.242 | 29113 | 2806 | 0.459 | 0.386 |
| 7 | 13.637 | 20594 | 1838 | 0.325 | 0.253 |
| Total |  | 6337641 | 726845 | 100.00 | 100.00 |


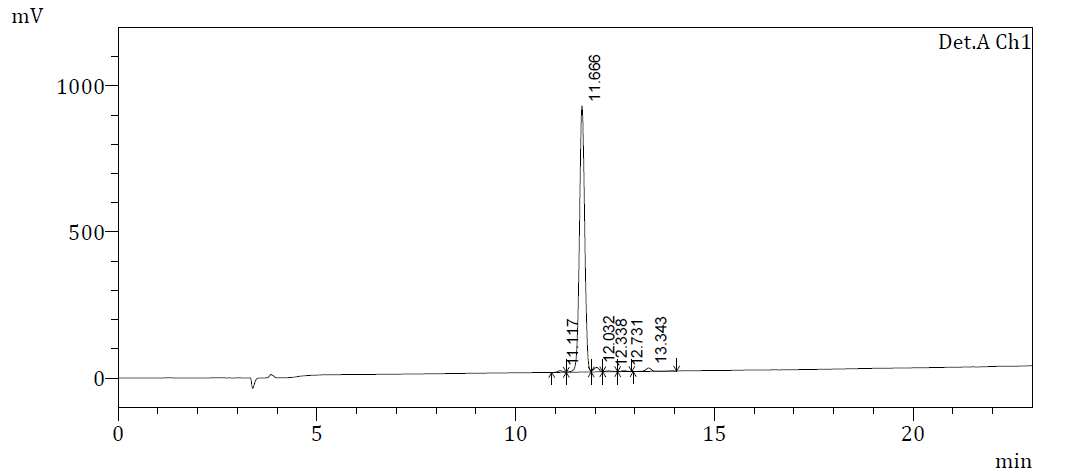


Fig. 3 HPLC chromatogram of B_3_ component

Table. 3 HPLC target peak information for B_3_ component

| Peak# | Ret. Time | Area | Height | Area % | Height % |
| --- | --- | --- | --- | --- | --- |
| 1 | 11.117 | 53385 | 5881 | 0.611 | 0.619 |
| 2 | 11.666 | 8347861 | 911335 | 95.475 | 95.894 |
| 3 | 12.032 | 148086 | 15520 | 1.694 | 1.633 |
| 4 | 12.338 | 39785 | 2994 | 0.455 | 0.315 |
| 5 | 12.731 | 30145 | 3208 | 0.345 | 0.338 |
| 6 | 13.343 | 124279 | 11422 | 1.421 | 1.202 |
| Total |  | 8743540 | 950360 | 100.00 | 100.00 |

S2, Original image of NF-κB; The molecular weight of the NF-κB protein is 60 kDa.


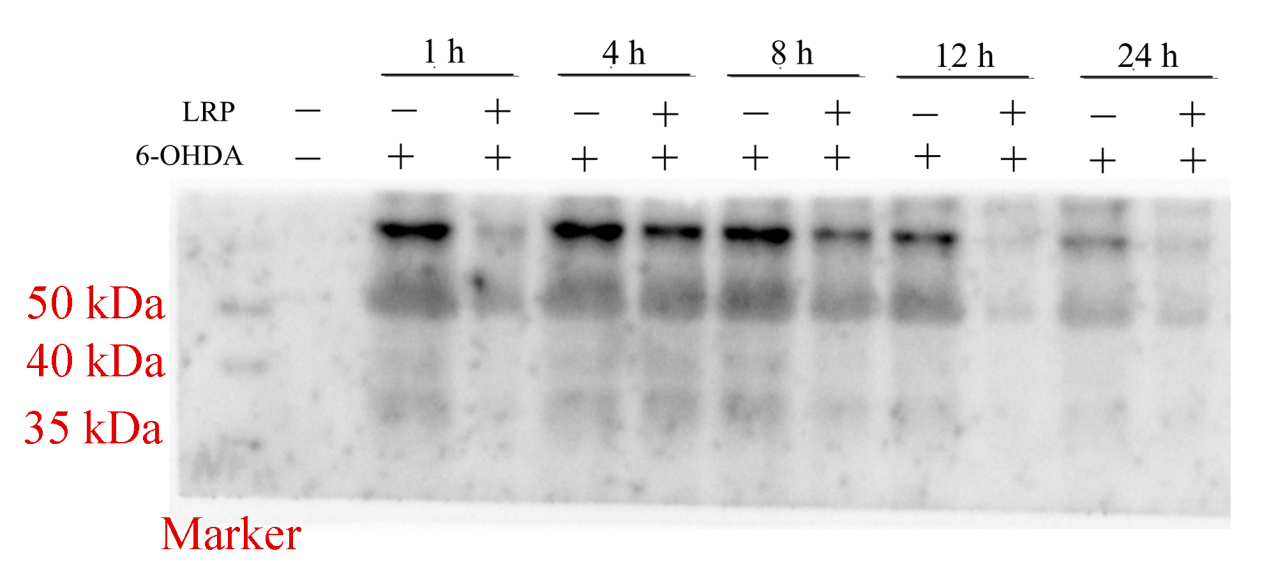


S3, Original image of LaminB for NF-κB; The molecular weight of the LaminB protein is 220 kDa. In this study, LaminB protein was determined to be an internal reference protein for NF-κB protein.


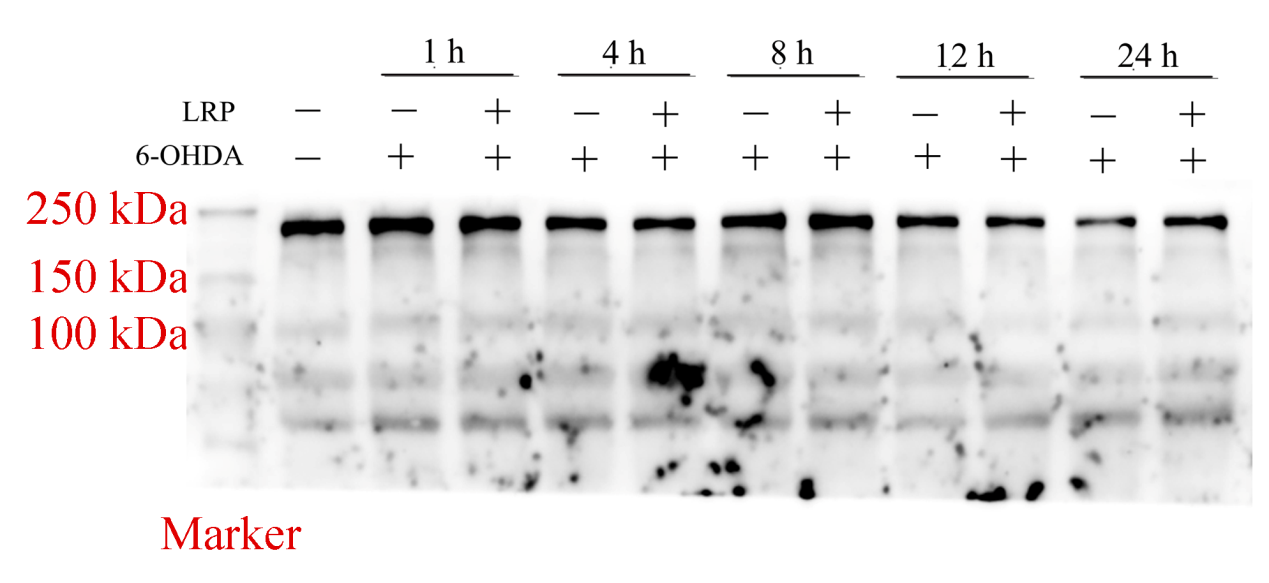


S4, Original image of IκBα; The molecular weight of the IκBα protein is 40 kDa.


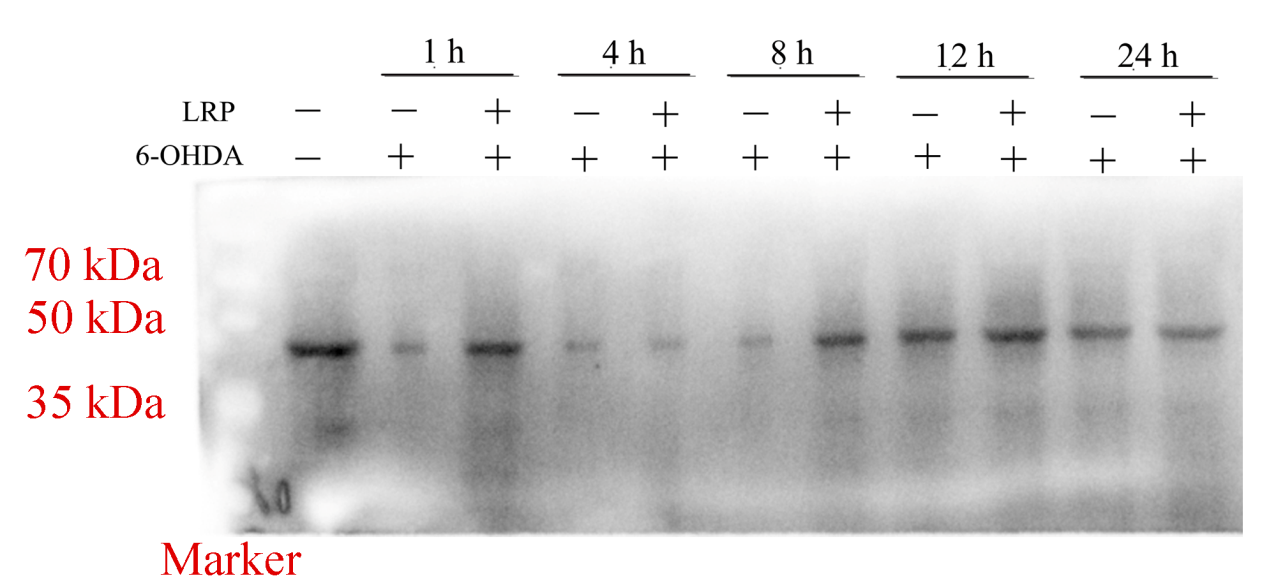


S5, Original image of p-IκBα; The molecular weight of the p-IκBα protein is 40 kDa.


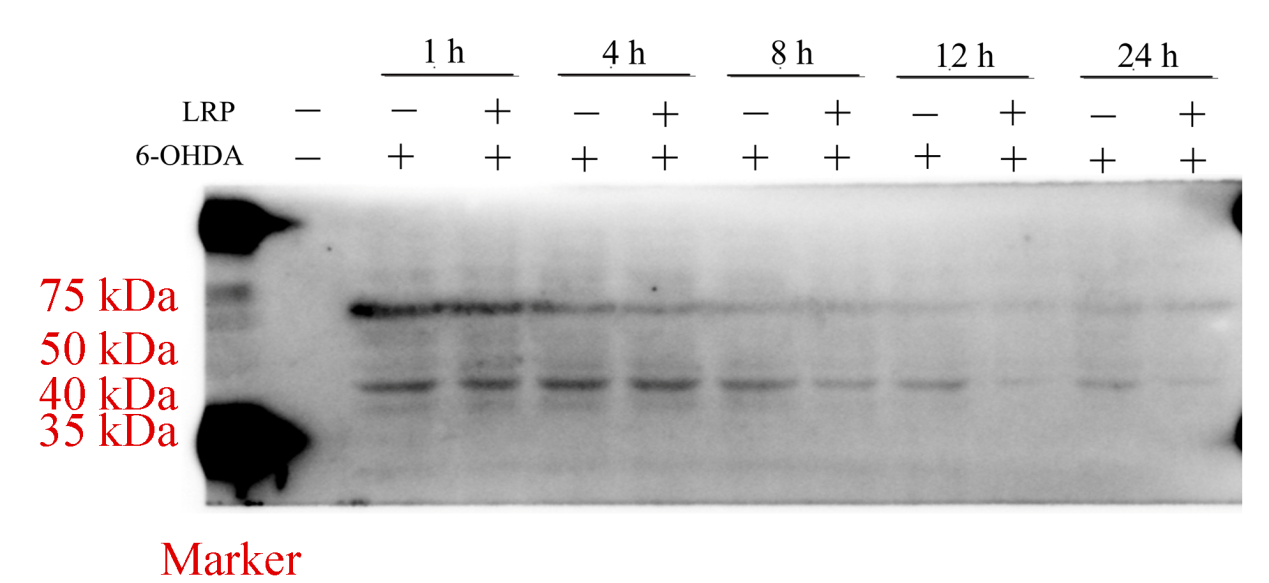


S6, Original image of GAPDH for IκBα and p-IκBα; The molecular weight of the GAPDH protein is 37 kDa. In this study, GAPDH protein was determined to be an internal reference protein for IκBα and p-IκBα protein.


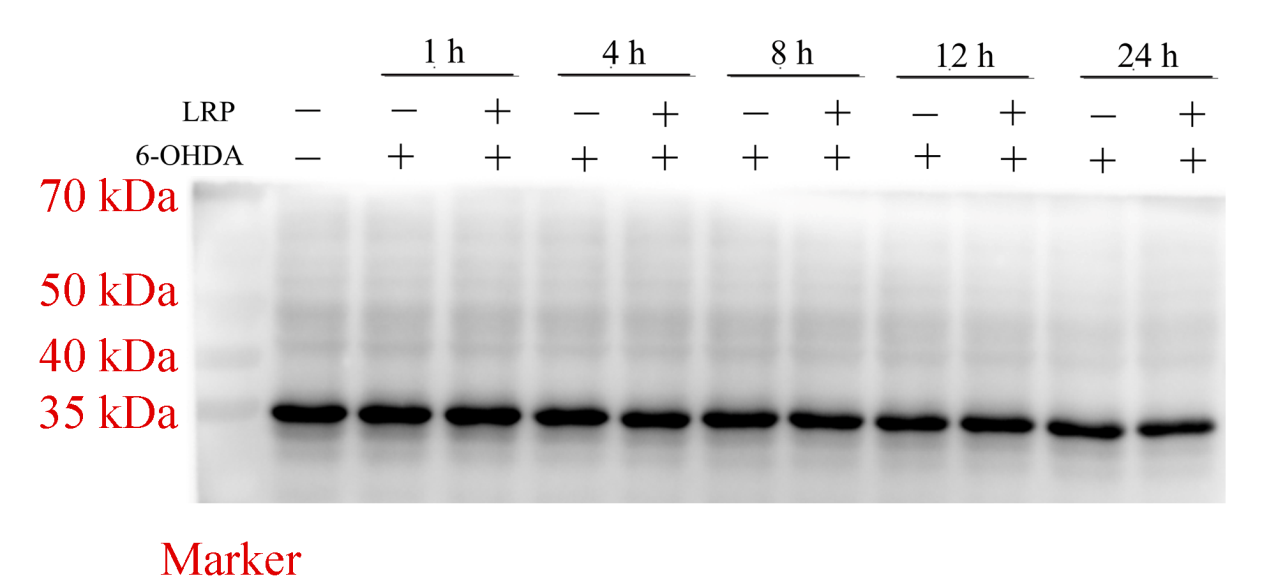


S7, Original image of IκBα in the nucleus; The molecular weight of the IκBαprotein in the nucleus is 40 kDa.


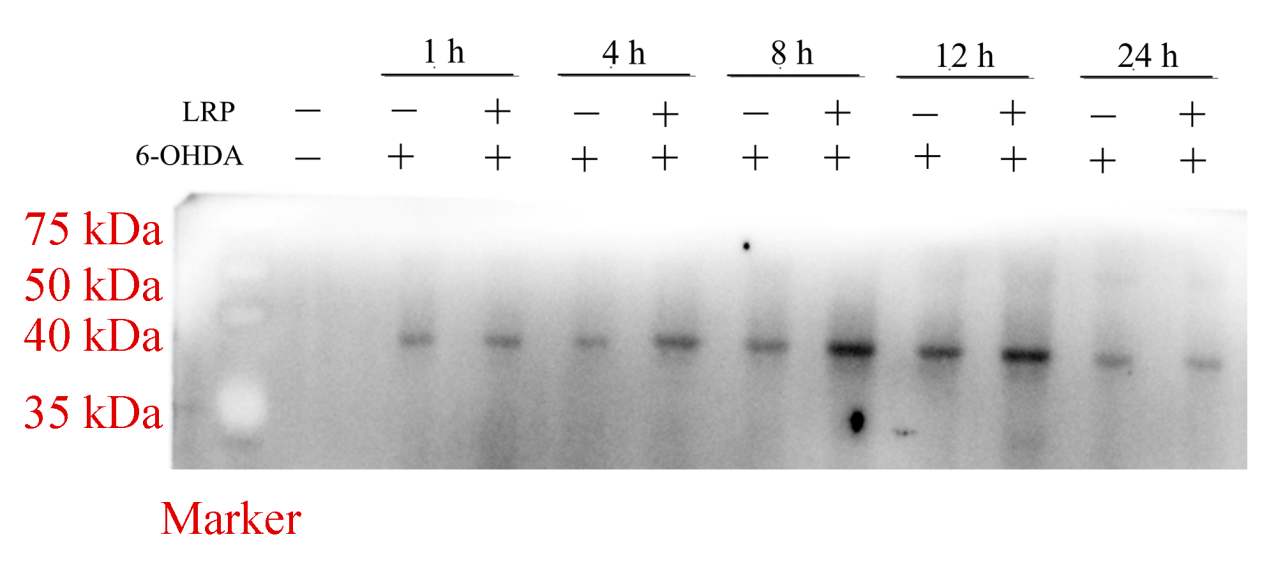


S8, Original image of LaminB for IκBα in nucleus; The molecular weight of the LaminB protein is 220 kDa. In this study, LaminB protein was determined to be an internal reference protein for IκBα protein in nucleus.


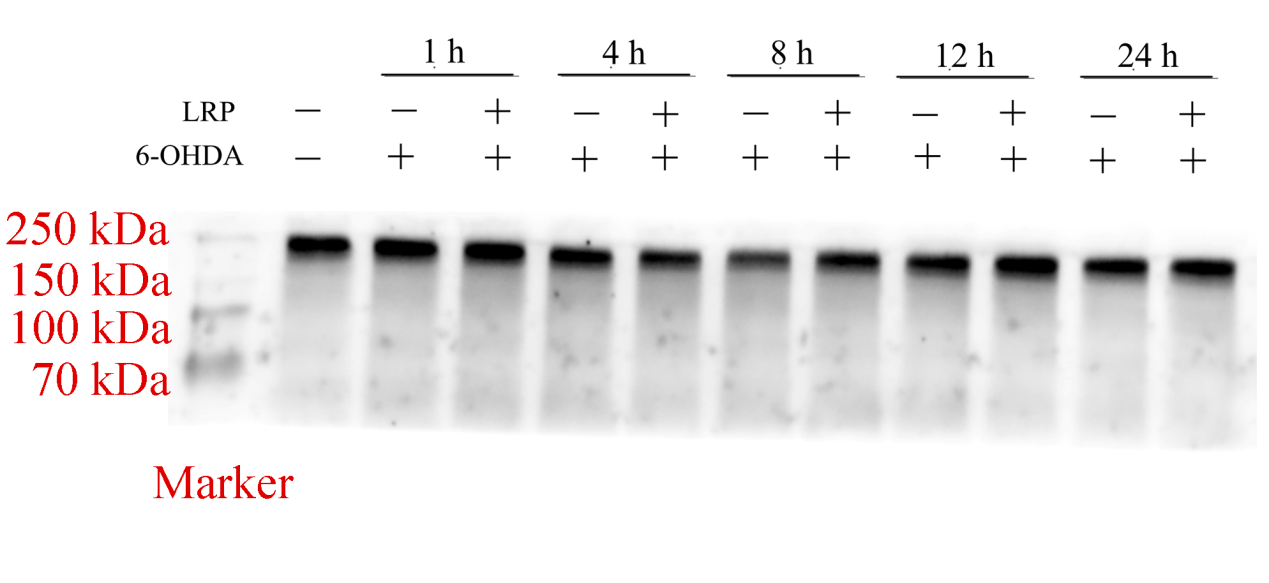

Supplement: Supplementary file 1 — Appendix S1 [file FSN3-11-2152-s001.docx]
